# Differential expression of colon cancer associated transcript1 (CCAT1) along the colonic adenoma-carcinoma sequence

**DOI:** 10.1186/1471-2407-13-196

**Published:** 2013-04-17

**Authors:** Bilal Alaiyan, Nadia Ilyayev, Alexander Stojadinovic, Mina Izadjoo, Marina Roistacher, Vera Pavlov, Victoria Tzivin, David Halle, Honguang Pan, Barry Trink, Ali O Gure, Aviram Nissan

**Affiliations:** 1 The Surgical Oncology Laboratory, Department of Surgery, Hadassah-Hebrew University Medical Center, Mount Scopus, POB 12000, Jerusalem, 91120, Israel; 2 Diagnostics and Translational Research Center Henry M Jackson Foundation for the Advancement of Military Medicine, Gaithersburg, MD, 20879, USA; 3 The Department of Surgery, Division of Surgical Oncology, Walter Reed National Medical Center, Bethesda, MD, USA; 4 Johns Hopkins School of Medicine, Baltimore, MA, USA; 5 Department of Molecular Biology and Genetics, Bilkent University, Ankara, Turkey; 6 Department of Surgery, Hadassah-Hebrew University Medical Center Ein Kerem, Jerusalem, Israel

**Keywords:** Colon cancer, Non-coding RNA, Biomarkers, Adenoma, Carcinoma

## Abstract

**Background:**

The transition from normal epithelium to adenoma and, to invasive carcinoma in the human colon is associated with acquired molecular events taking 5-10 years for malignant transformation. We discovered CCAT1, a non-coding RNA over-expressed in colon cancer (CC), but not in normal tissues, thereby making it a potential disease-specific biomarker. We aimed to define and validate CCAT1 as a CC-specific biomarker, and to study CCAT1 expression across the adenoma-carcinoma sequence of CC tumorigenesis.

**Methods:**

Tissue samples were obtained from patients undergoing resection for colonic adenoma(s) or carcinoma. Normal colonic tissue (n = 10), adenomatous polyps (n = 18), primary tumor tissue (n = 22), normal mucosa adjacent to primary tumor (n = 16), and lymph node(s) (n = 20), liver (n = 8), and peritoneal metastases (n = 19) were studied. RNA was extracted from all tissue samples, and CCAT1 expression was analyzed using quantitative real time-PCR (qRT-PCR) with confirmatory *in-situ* hybridization (ISH).

**Results:**

Borderline expression of CCAT1 was identified in normal tissue obtained from patients with benign conditions [mean Relative Quantity (RQ) = 5.9]. Significant relative CCAT1 up-regulation was observed in adenomatous polyps (RQ = 178.6 ± 157.0; p = 0.0012); primary tumor tissue (RQ = 64.9 ± 56.9; p = 0.0048); normal mucosa adjacent to primary tumor (RQ = 17.7 ± 21.5; p = 0.09); lymph node, liver and peritoneal metastases (RQ = 11,414.5 ± 12,672.9; 119.2 ± 138.9; 816.3 ± 2,736.1; p = 0.0001, respectively). qRT-PCR results were confirmed by ISH, demonstrating significant correlation between CCAT1 up-regulation measured using these two methods.

**Conclusion:**

CCAT1 is up-regulated across the colon adenoma-carcinoma sequence. This up-regulation is evident in pre-malignant conditions and through all disease stages, including advanced metastatic disease suggesting a role in both tumorigenesis and the metastatic process.

## Background

Colon carcinoma (CC) is a common disease affecting over a million people annually worldwide [1,2]. Major advances in multi-modality therapy for CC over the past decade have amounted to improved survival [3-5]. The ability to identify, validate and apply clinically novel disease-specific biomarkers may improve diagnostic accuracy, disease staging, patient follow up and treatment selection, and biomarkers stand to advance further positive treatment-related outcomes.

There are no clinically useful biomarkers currently in widespread use for the diagnosis of CC. A stool-based molecular assay for diagnosis was shown in a recent study to have high diagnostic sensitivity and specificity for CC [6,7]. Two tumor-related biomarkers used as an adjunct to staging as well as for post-treatment surveillance for disease recurrence are Carcino- Embryonic Antigen (CEA) and Carbohydrate Antigen 19-9 (CA-19-9) [8,9]. Neither CEA nor CA19-9 is sufficiently sensitive or specific for CC staging or post-treatment surveillance [10,11].

A large number of genetic and epigenetic alterations have been studied as potential biomarkers intended to enable early disease detection, optimize cancer staging, and facilitate accurate estimation of prognosis in CC [12,13]. There is an increasing number of microRNA fragments found in CC primary tumor tissues, metastasis, and plasma [14-17] that may serve as biomarkers for the detection of CC, estimating prognosis, and use in the follow up of CC patients to assess treatment response and disease state [18]. Changes in DNA methylation patterns of specific genomic regions are considered to be among the most common molecular alterations in CC [19,20]. The transcriptome of CC has also been studied showing about 0.5% of protein-coding gene transcripts up-regulated in tumor tissue compared to normal tissue [21]. Importantly, another transcriptomic study identified one transcript that was over expressed as much as 50-fold in CC over normal tissues [22]. Despite advances in high throughput proteomic characterization of aberrant protein expression and disease-specific differentiation from normal colonic tissue, very few biomarkers have been found to be clinically useful and have attained widespread clinical application [23,24].

Colon Cancer Associated Transcript 1 (CCAT1) is a 2628 nucleotide-long, non-coding RNA recently discovered using Representational Difference Analysis (RDA), cDNA cloning, and rapid amplification of cDNA ends (RACE) [25]. CCAT1 is located in the vicinity of c-MYC, a well-known transcription factor. Preliminary experiments showed CCAT1 up-regulation in tumor cell lines and tissues obtained from CC patients. Studies in human tissues showed minimal expression in normal liver and small bowel tissue; however, no CCAT1 expression was detected in many other human tissues tested. The location of CCAT1 on chromosome 8q24.21 is significant since this area was described before as a “hot spot” harboring multiple genetic alternations in both colon and prostate cancer [26,27].

The current study was based on initial exploratory findings of increased CCAT1 expression in colon adenocarcinoma but extremly low transcript expression in normal human tissue [25]. The principal aim of this study was to further characterize expression of this novel molecular marker for CC. CCAT1 expression is investigated across the spectrum of CC carcinogenesis in the current study: from normal tissues, through adenoma, as well as invasive carcinoma, to include lymph node as well as distant metastasis.

## Methods

### Patients

This is a prospective pathological and molecular study of primary colon and appendecial adenoma, adenocarcinoma, regional nodal tissue and distant metastasis (liver and peritoneal) from patients undergoing resection of the primary tumor, regional lymph nodes, and/or metastasis, as well as patients undergoing colon resection for benign conditions. The study was approved by the Institutional Review Board (Helsinki Committee; Protocol 391-04-08-06). Patients with a diagnosis of primary, or metastatic (Clinical UICC-AJCC Stage I-IV) CC or patients scheduled to undergo colon resection for benign conditions, were included. To be eligible for study patients had biopsy-proven, primary CC or adenoma, had a benign condition requiring colon resection, were >18 years-of-age, and capable of providing informed consent. Written informed consent was obtained from all participants.

### Tissue procurement

Tissues were obtained from random areas of the resected colon of patients with benign conditions, from the primary lesion (adenoma or invasive adenocarcinoma), and from normal appearing mucosa adjacent to the primary tumor site. Lymph nodes were obtained from the mesocolon after sufficient tissue was submitted for standard histopathological analysis. Liver metastasis were obtained from patients undergoing hepatic resection and peritoneal metastasis were obtained from patients undergoing cytoreductive surgery and hyperthermic intra-peritoneal chemotherapy. Immediately following surgical resection, the specimen was delivered fresh to the Department of Pathology, where, under the supervison of an attending pathologist, a small portion of resected tissue was snap frozen in liquid nitrogen for future RNA extraction. One hundred twenty tissue samples from 94 study subjects were collected. Seven patients [(peritoneal (n = 6), and liver metastases (n = 1)] were excluded because RNA extracted from their tissue was of low quality, insufficient for qRT-PCR, leaving 113 tissue samples from 87 patients for analysis.

### Total RNA isolation from tissues

Total RNA was extracted using the miRvana® isolation kit (Ambion Inc., Austin, TX) in accordance with manufacturer instructions. Weighed tissues were thoroughly crushed on dry ice and disrupted with 1 ml/50-100 mg tissue, denaturizing lysis buffer using a polytron tissue homogenizer. RNA concentration was measured with NanoDrop® Spectrophotometer (ND-100, NanoDrop Technologies, Wilmington, DE) and stored at -80°C until further use.

### Synthesis of cDNA

Following DNase treatment, cDNA synthesis was performed using random primer (Roche Diagnostics GmbH, Mannheim, Germany) added to 10 μl of RNA. After incubation, 1 μl of reverse transcriptase (SuperScript II Reverse Transcriptase 200 U/μl, Invitrogen, Carlsbad, CA) was added. The cDNA was stored at -20°C until used for qRT-PCR.

### Real time quantitative PCR

Primers used were: CCAT1 (custom designed by Applied Biosystems Inc., Foster City, CA):

CCAT1-Forward – TCACTGACAACATCGACTTTGAAG

CCAT1-Reverse - GGAGAAAACGCTTAGCCATACAG

CCAT1-Probe - Fam-TGGCCAGCCCTGCCACTTAC- ZNA-4-BAQ-1

GAPDH (probe dye: VIC-MGB 4326317E-0411007) was used as a control gene.

CCAT1 RNA was normalized to GAPDH-RNA content using ABI 7500 SDS software, v1.2.2 (Applied Biosystems Inc., Foster City, CA). Positive and negative controls, as well as samples with no DNA were included in every qRT-PCR experiment. PCR reactions were performed using ABI qRT-PCR thermocycler (7500 Real Time PCR System, Applied Biosystems Inc., Foster City, CA). The qRT-PCR program was run for 40 cycles, following an initial incubation at 95°C, 10 min. Each cycle consisted of 95°C × 15 sec. and 60°C × 1 min.

### *In situ* hybridization

Fluorescein isothiocyanate (FITC) labeled CCAT1 probe was used for *in situ* detection of CCAT1 in formalin fixed paraffin embedded (FFPE) colon tissues in accordance with a standardized protocol [28]. Briefly, the de-paraffinized colon tissue slides were treated with protein K (24 g/ml) for 30 min. at room temperature. After washing with water, the slides were hybridized with 600 nM CCAT1 probe at 55°C for 90 min in a humidity chamber. The slides were then washed in Tris-Buffered Saline Tween-20 (TBST) for 25 min at 55°C with agitation to remove excess CCAT1 probe. Pre-diluted AP conjugated Anti-FITC antibody (Santa Cruz Biotechnology, Inc., Santa Cruz, CA) was applied onto the tissue samples for 30 min at room temperature followed by color development using 5-Bromo-4-chloro-3-indolyl phosphate (BCIP) as a substrate.

### Statistical analysis

Summary statistics were obtained using established methods. Associations between categorical factors were studied with Fisher’s exact test or Chi-squared test, as appropriate. Continuoues variables between study groups were compared using the *T*-test (two-sided). Statistical analysis was performed using IBM-SPSS® statistical package Version 19.0 (SPSS Inc. Chicago, IL). A p value < 0.05 was considered significant.

## Results

Tissue samples were obtained from patients (n = 94) undergoing surgery for benign inflammatory conditions, adenomatous polyps or various stages of CC. In patients with distant metastatic disease (n = 34), one liver (1/9) and six (6/25) peritoneal metastases were excluded as indicated above. Overall, RNA was successfully extracted from 113 of 120 samples (94.2%) obtained from 87 patients and found to be suitable for analysis.

### CCAT1 expression in benign inflammatory colonic tissues

RNA was extracted from patients with various non-malignant conditions (n = 10, Table 1). Using comercially available normal colonic RNA as a calibrator, mean CCAT1-RQ was 5.9 ±5.6. Compared to normal colonic RNA, there was 1-5 fold up-regualtion of CCAT1 expression in 70% (7/10) of inflammed colonic tissue. Interestingly, in three of these 7 patients, one with perforated appendicitis and an inflammatory mass, and two with severe complicated diverticulits requiring emergent surgical intervention, inflammatory colonic tissue CCAT1 was expressed to an even greater degree: 11-13 fold relative to normal colonic tissue RNA.

**Table 1:** Clinical and pathological charactaristics of patients with inflammatory conditions participating in the study

**N**	**Sample number**	**Diagnosis**	**Age**	**Gender**	**Procedure**	**RQ**
1.	814	Ischemic colitis	47	Male	Subtotal colectomy	2.02
2.	595	Perforated appendecitis	31	Male	RT colectomy	15.338
3.	827	Diverticulitis	73	Male	Sigmoid resection	14.929
4.	854	Diverticulitis	33	Male	Sigmoid resection	13.881
5.	331	Diverticulitis	64	Female	Sigmoid resection	3.004
6.	351	Perforated volvulus	69	Male	Sigmoid resection	2.154
7.	537	Diverticulitis	49	Female	Sigmoid resection	1.162
8.	594	Diverticulitis	54	Female	Sigmoid resection	5.54
9.	704	Diverticulitis	36	Male	Sigmoid resection	1.402
10.	803	Diverticulitis	67	Female	Sigmoid resection	4.88
11.	Colon NN Ambion®	* Trauma			unknown	1

*RQ* = Relative Quantity of CCAT-1 RNA.

* commercially available RNA mixture.

### CCAT1 expression in normal colonic mucosa adjacent to the primary colon adenocarcinoma

**Figure 1:**
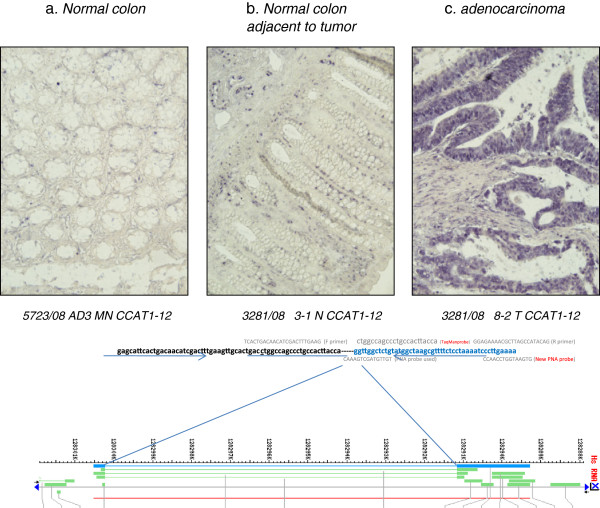
**CCAT1 expression analyzed by *in-situ *hybiridization.***In situ* hybrdization of CCAT1 was analyzed in normal colonic tissue (**a**), normal mucosa adjacent to the primary tumor site (**b**) and in primary adenocarcinoma of the colon (**c**). Samples **a** and **b** were obtained from the same patient (T877). The probe sequence and topographic location within the CCAT1 gene are outlined in the lower part of the figure.

In a previous study [22], we obserevd high levels of CCAT1 expression in histologically normal appearing colonic mucosa obtained from patients with primary CC. We therefore analyzed normal-appearing mucosa sampled in the vicinity of the tumor in 16 of 22 patients with primary CC. Mean (±SD) RQ for normal tisssues was 17.7 ± 21.5. Significant CCAT1 up-regualtion (>10% of tumor tissue) was observed in 63% (10/16) of peri-tumoral normal tissue. To rule out contamination of normal mucosa by cancer cells shed at time of surgery or tissue handling after CC resection, we studied CCAT1 expression in tumor and adjacent normal tissues by *in-situ* hybridization and compared CCAT1 expression intensity to normal colonic tissue obtained from patients with no known colonic disease operated for trauma. The qRT-PCR results obtained in this study were confirmed by *in-situ* hybridization staining (Figure 1), thereby making contamination as a source of false positive finding very unlikely.

### CCAT1 expression in adenomatous polyps

**Figure 2:**
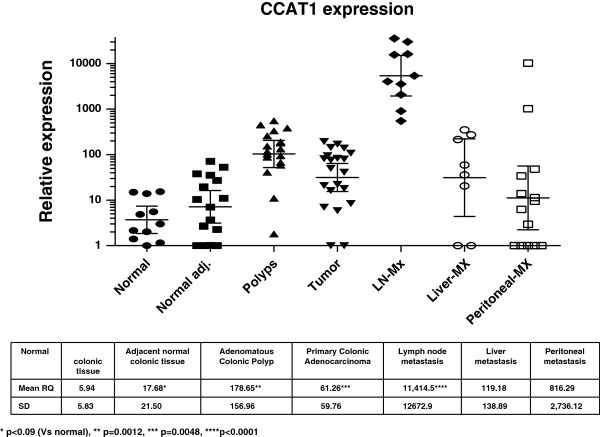
**A logarithmic scale of relative quantity (RQ) of CCAT1 expression in the adenoma-carcinoma sequence.** The log RQ of CCAT1 expression is shown for every tissue type: inflamed colonic tissue, normal colon adjacent to the tumor site (Normal Adj.), adenomatous polyps, primary tumor tissue, lymph node metastasis (LN-Mx), liver metastasis (Liver-Mx), and peritoneal metastasis (Peritoneal-Mx). Mean RQ and standard deviation are shown in the lower part of the figure. Mean CCAT1 expression was compared to normal colon. * p < 0.09, ** p = 0.012, ***p = 0.0048, ****p < 0.0001.

Patients with adenomatous polyps (n = 18) >10 mm in size who failed endoscopic resection, underwent colectomy. Clinical and histopathological details are outlined in Table 2. There were 6 (33%) tubular adenomas, 2 (11%) villous adenomas, and 10 (56%) tubulovillous adenomas. Low- and high-grade dysplasia was diagnosed in 5 (28%) and 13 (72%) patients, respectively. No statistically significant correlation was observed between adenoma sub-type (p = 0.24) or degree of dysplasia (p = 0.68), and CCAT1 expression. Mean CCAT1 RQ was 176.9 ± 148.7. CCAT1 was significantly (>10 fold) up-regulated in 17 of 18 (94%) adenomatous polyps studied. Of these 17 positive samples, CCAT1 had very high expression (>100 fold) in 11 of 18 (61%). The difference between normal (inflammatory) tissues and pre-malignant tissues is demonstrated in Figure 2.

**Table 2:** CCAT1 expression in adenomatous polyps

**Sample number**	**Diagnosis**	**Dysplasia**	**Age**	**Gender**	**RQ**
1016P1	TA	LGD	66	F	102.32
1030P1	TVA	HGD	40	M	154.34
1052P1	TA	HGD	75	F	258.50
1065P	VA	HGD	75	M	11.03
1079P	TA	HGD	23	M	1.80
597P	TVA	HGD	40	F	116.08
608P2	TVA	HGD	64	M	132.88
619P	TA	LGD	72	F	40.93
626P	TVA	HGD	33	M	94.42
760P9	TVA	HGD	73	M	180.52
778P	TVA	HGD	64	F	67.65
809P	TVA	HGD	71	F	189.49
844P1	TVA	HGD	78	M	58.57
872P	TA	HGD	67	F	382.15
881P	TVA	HGD	73	F	334.07
887P1	TVA	HGD	65	M	554.10
932P	VA	LGD	58	F	88.04
935P1	TA	LGD	72	M	448.82

*RQ* = Relative Quantity of CCAT1 RNA.

*TA* = tubular adenoma, *VA* = villous adenoma, *TVA* = tubulovillous adenoma.

*LGD* = low grade dysplasia, *HGD* = high grade dysplasia.

*M* = male, *F* = female.

IMC = intra-mucosal carcinoma.

### The expression of CCAT1 in primary tumor tissue of patients with adenocarcinoma of the colon

As CCAT1 was first shown to be up-regulated in human CC tissue [22], we anlayzed a new patient cohort with AJCC Stage I-III CC (n = 22, Table 3). Mean RQ for tumor tisssues was 64.9 ± 56.9. There were 12 female patients with slightly higher values of CCAT1 expression (RQ = 69.2 ± 64.5) than that found in male patients (RQ = 41.4 ± 39.2; p = 0.29). There was higher expression of CCAT1 in patients ≥ 60 years of age (RQ = 75.8 ± 72.8 vs. 40.4 ± 36.5 for patients <60 years of age, p = 0.17). There was no statistically significant correlation between T-Stage, N-Stage or AJCC Stage and CCAT1 expression. Further, primary tumor grade, mucin production, lympho-vascular or perineural invasion did not correlate significantly with CCAT1 expression in primary CC. There was, however, a trend toward higher CCAT1 expression in right-sided (n = 9; RQ = 81.7 ± 74.1) versus left-sided tumors (n = 13; RQ = 42.2 ± 42.3, p = 0.13). Overall, CCAT1 up-regulation of 5-fold or higher compared to normal colon was seen in 20/22 (90.1%) of CC samples.

**Table 3:** Anatomical and Histological characteristics of tumer samples

**Sample**	**Anatomic location**	**AJCC stage**	**Grade**	**Mucin**	**LVI**	**BVI**	**Neural**	**Age (years)**	**Gender**	**RQ-T**	**RQ-N**
**612**	**LT**	**3**	**2**	**NO**	**NO**	**NO**	**NO**	**70.00**	**F**	**7.01**	**71.26**
**655**	**LT**	**3**	**2**	**NO**	**NO**	**NO**	**NO**	**73.00**	**F**	**5.91**	**0.021**
**662**	**LT**	**3**	**2**	**NO**	**NO**	**NO**	**NO**	**71.00**	**M**	**18.15**	**2.65**
**681**	**LT**	**3**	**2**	**NO**	**NO**	**NO**	**NO**	**54.00**	**F**	**50.24**	
**698**	**LT**	**3**	**2**	**NO**	**NO**	**NO**	**NO**	**60.00**	**M**	**8.46**	
**712**	**LT**	**3**	**2**	**NO**	**NO**	**NO**	**YES**	**40.00**	**M**	**83.11**	**52.60**
**759**	**LT**	**2**	**2**	**NO**	**NO**	**NO**	**NO**	**71.00**	**M**	**16.33**	**10.345**
**760**	**RT**	**3**	**2**	**YES**	**NO**	**NO**	**NO**	**73.00**	**M**	**21.01**	**2.27**
**766**	**LT**	**3**	**2**	**NO**	**NO**	**NO**	**NO**	**70.00**	**F**	**139.49**	**7.04**
**781**	**RT**	**2**	**2**	**NO**	**NO**	**NO**	**NO**	**42.00**	**F**	**82.03**	**0.35**
**809**	**RT**	**2**	**2**	**YES**	**NO**	**NO**	**NO**	**70.00**	**F**	**171.37**	**1.01**
**828**	**LT**	**4**	**3**	**NO**	**YES**	**YES**	**YES**	**53.00**	**M**	**22.15**	**35.24**
**829**	**LT**	**3**	**2**	**NO**	**NO**	**NO**	**NO**	**46.00**	**M**	**94.81**	**27.15**
**838**	**RT**	**2**	**2**	**YES**	**NO**	**NO**	**NO**	**46.00**	**M**	**0.00**	
**844**	**RT**	**2**	**2**	**YES**	**NO**	**NO**	**NO**	**78.00**	**M**	**108.38**	**3.68**
**853**	**LT**	**3**	**2**	**NO**	**NO**	**NO**	**NO**	**50.00**	**F**	**0.00**	
**861**	**RT**	**3**	**2**	**NO**	**NO**	**NO**	**NO**	**74.00**	**F**	**194.12**	
**881**	**RT**	**3**	**3**	**NO**	**YES**	**NO**	**YES**	**72.00**	**F**	**76.37**	**0.99**
**883**	**LT**	**3**	**2**	**NO**	**NO**	**NO**	**NO**	**53.00**	**F**	**41.41**	**19.55**
**887**	**RT**	**2**	**2**	**NO**	**NO**	**NO**	**NO**	**56.00**	**F**	**80.45**	**37.77**
**905**	**LT**	**2**	**2**	**NO**	**NO**	**NO**	**NO**	**53.00**	**F**	**62.21**	**11.03**
**1013**	**RT**	**2**	**2**	**NO**	**NO**	**NO**	**NO**	**87.00**	**M**	**145.11**	

*AJCC* = American Joint Committee on Cancer.

*BVI* = blood vessel invasion, *LVI* = lymph vessel invasion, neural = neural invasion.

*Mucin* = presence of mucin within or around the tumor.

*RQ-T* = relative quantity of CCAT1 expression in tumor tissue.

*RQ-N* = relative quantity of CCAT1 expression in adjacent normal tissue.

*M* = male, *F* = female.

### The expression of CCAT1 in lymph node metastasis of patients with adenocarcinoma of the colon

**Figure 3:**
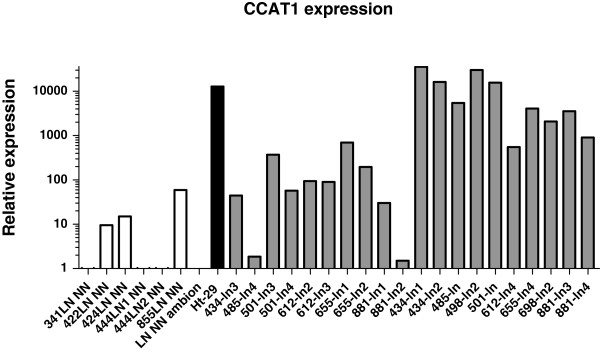
**A logarithmic scale of relative quantity (RQ) of CCAT1 expression in lymph nodes.** The log RQ of CCAT1 expression is shown for benign lymph nodes (white rectangle), lymph nodes without metastasis by histopatholgical examination (gray rectangle), and for lymph nodes with metastasis from the same colon cancer patients (black rectangle). The colon cancer cell line HT29 was used as positive control and a commercially available normal lymphatic tissue was used as a calibrator (LN_NN-Ambion).

Lymph nodes (LNs) from patients undergoing surgery for CC (n = 10) were sectioned in half, with one half of the node undergoing standard histopathological examination, and the other half of the node snap frozen for subsequent analysis. There was no CCAT1 expression in the LNs obtained from patients with benign inflammatory conditions (n = 6, Figure 3). CCAT1 was highly up-regulated in all metastatic LNs having a mean RQ = 11,414.5 ± 12,672.9 compared to a mean RQ of 12.4 ± 21.3 for benign LNs (p = 0.019) and compared to lymph nodes not harboring metastasis by histopatholgical examination obtained from the same patients (n = 10). The RQ was 157.2 ± 218.2 for the histologically benign LNs (p = 0.021). In three patients (patients #612, #655, and #698) we had matched tissues of primary tumor and lymph node metastasis. CCAT1 expression was up-regulated in all three primary tumors (Table 3).

### CCAT1 expression in liver metastasis of colorectal cancer origin

Representative tissue samples obtained from resected CC liver metastasis were analysed (n = 8). All samples were obtained from patients treated with systemic therapy before resection of liver metastasis. CCAT1 was up-regulated in 6 of 8 (75%) of tissues studied. Mean RQ for normal tisssues was 119.2 ± 138.9.

### CCAT1 expression in peritoneal metsastasis of colorectal carcinoma and appendiceal neoplasms

Peritoneal spread of CC may have a different mechanism of dissemination than that of visceral CC metastasis. Adenocarcinoma and other neoplasms originating from the vermiform appendix may also have both different pathogenesis and different molecular features than CC. Therefore, we elected to study CCAT1 expression in tissues obtained from 19 patients with peritoneal metastasis of colon (n = 14) and appendiceal origin (n = 5). All patients received systemic therapy before cytoreductive surgery. There was low CCAT1 expression in 20% (1/5; mean RQ = 0.99 ± 1.9) of appendiceal neoplasms (4 adenocarcinomas and one pseudomyxoma peritonei), while it was upregulated in 50% (7/14; mean RQ = 816.3 ± 2,736.1) of peritoneal metastasis of colonic origin. This difference was not statistically different (p = 0.21) mainly due to large varibility in the CCAT1 expression of peritoneal metastasis of colonic origin.

## Discussion

The sequential progression of colon tumorigenesis provides an excellent system to assess promising biomarkers for colon cancer screening and early detection. This adenoma-carcinoma sequence leading to CC is well described, and is characterized by multiple genetic and epigenetic events [29]. However, to the best of our knowledge, there is not a single molecule uniformly up-regulated in all phases of CC development.

Colon Cancer Associated Transcript 1 (CCAT1) is a unique transcript up-regulated in CC compared to normal human tissues [25]. Its role in tumorigenesis has yet to be defined. Previous observations demonstrated up-regulation of CCAT1 in over 90% of colonic adenocarinomas, with relatively little or no transcript expression in a panel of normal human tissues [25]. We identified slightly higher expression of CCAT1 in colonic tissues obtained from patients with benign colonic disorders compared to normal, non-inflamed colonic tissue. This slight up-regulation was more prominent in the tissue samples of patients with inflammatory conditions and reached a 15-fold increase in patients with severe colonic inflammation. If this observation can be replicated in chronically inflamed colonic tissues such as those obtained from inflammatory bowel disease patients, this may suggest a role for CCAT1 in neoplastic transformation often observed in chronically inflamed tissues.

In this study we have shown again that CCAT1 is up-regulated in most (20/22) primary tumor tissues. Interestingly, CCAT1 was also up-regulated, albeit to a lesser degree, in many of the histologically normal appearing mucosa samples adjacent to the primary tumor site. This may be caused by a contamination of nearby cancer cells shed during surgery or during tissue handling. In to order address this potential issue of cellular contamination, we used formalin fixed paraffin-embedded tissue of one of the study subjects showing CCAT1 up-regulation in the primary tumor tissue and to a lesser degree in the adjacent normal tissue (T877) by qRT-PCR, and analyzed transcript expression by *in-situ* hybridization. Good correlation was observed between the qRT-PCR findings and the *in-situ* hybridization findings indicating that this is a real biological phenomenon rather than a false positive finding related to local tumor cell contamination. Currently we are in the process of performing *in-situ* hybridization on a large cohort of colon cancer patients to further study this phenomenon.

The up-regulation of a tumor marker in histologically normal-appearing tissue is a complex matter, a “double-edged sword.” On the one hand, this finding may provide a powerful tool to predict future risk of colon cancer by studying biomarker expression in random colonic mucosal biopsies in screening or high-risk populations. On the other hand, biomarker expression in clinically disease-free patients may be interpreted as a false positive finding, which amounts to reduced diagnostic accuracy of the test. If co-expression of CCAT1 and DNA-methylation abnormalities shown to appear in early phases of the adenoma-carcinoma sequence [30] can be found, this co-expression may be more predictive of adenoma-carcinoma sequence progression, and will serve as the basis for development of risk reduction or early curative intervention strategies.

A clinically important stage in the adenoma-carcinoma process is the formation of an adenomatous polyp. We analyzed tissue from adenomatous polyps for CCAT1 expression. We restricted our analysis to polyps larger than 10 mm in diameter, in order not to compromise histopathological diagnosis. The *in-situ* technique for CCAT1, described earlier [28], may provide a tool for future analysis of CCAT1 expression in smaller polyps, and for differential diagnosis of hyperplastic and adenomatous polyps, namely those with uniformly benign and those with possibly malignant natural history. We observed CCAT1 to be up-regulated in all but one of the 18 adenomatous polyps studied. In 61% of these adenomatous polyps the transcript expression exceeded 100-fold relative to that of normal colon tissue. This observation provides supportive evidence of a role of CCAT1 in the early neoplasia (adenoma formation) stage of colon carcinoma pathogenesis. The finding of mean CCAT1 expression in adenoma significantly exceeding that of carcinoma further supports this hypothesis, as it points to a down-regulating effect on CCAT1 expression once malignant transformation is attained.

CCAT1 up-regulation of 5-fold or higher compared to normal colon. Transcript up-regulation was seen in 90.1% of malignant primary tumor samples obtained from patients with Stage I-III colon adenocarcinoma. The fact that this non- coding RNA is located on chromosome 8q24.21, a “hot spot” for many cancer-related single nucleotide polymorphisms (SNPs), supports a role for CCAT1 in the tumorigenesis of colon carcinoma.

Current histopathological nodal staging techniques may overlook occult lymph node metastases amounting to pathological under-staging and under-treatment. Many investigators have tried to improve upon lymph node staging in patients with colon cancer [31-34]. We analyzed lymph nodes from patients with colon cancer having obvious macro-metastasis by standard histopathological staging for CCAT1 expression and compared this expression to that of negative lymph nodes by histopathology obtained from the same patients and to that of benign lymph nodes for patients without colon cancer. CCAT1 was highly up-regulated (over 100 fold) in all 10 metastatic lymph nodes studied. Such exceedingly high expression of CCAT1 may suggest an important role of this unique non-coding RNA in regional lymphatic and nodal dissemination of colon adenocarcinoma. Furthermore, this finding may be applied clinically for the detection of occult metastatic disease in seemingly disease-free regional lymph nodes of patients undergoing surgical resection of colon cancer with curative intent. This would improve staging accuracy and individualized treatment planning, specifically adjuvant systemic therapy in patients with nodal disease.

Two of the most common sites of metastatic spread of colon adenocarcinoma are the liver and peritoneum. Therefore, we included patients operated on for treatment of metastatic disease to these organ sites in our study. Unfortunately, all patients were previously treated by systemic therapy; therefore, treatment-related alterations in CCAT1 expression cannot be excluded in these pre-treated patients. However, the practical reality is that access to tissue of naïve (previously untreated) patients with colon cancer metastatic to the liver or peritoneum is limited, as it is a distinctly rare clinical scenario since most patients are treated, according to our evidence-based guidelines, with systemic therapy before surgery for metastatic disease.

Taking this potential bias into account, we showed that CCAT1 was up-regulated in liver as well as in peritoneal metastasis of colon cancer patients. The variability between the results may be due to the therapeutic efficacy of the previous chemotherapy, or may reflect true biological variability in CCAT1 expression. The only way to study this definitively is to obtain metastatic tissue before and after systemic therapy administration and demonstrate a decrease in CCAT1 expression in systemic treatment responders. Another non-coding RNA up-regulated in liver metastasis as well as in many cancer types is H19 [35]. Interestingly, its expression was also shown to be higher in histologically normal-appearing liver surrounding metastasis [36]. This correlates, in part, with our observation of CCAT1 up-regulation in normal colonic tissues adjacent to the primary tumor site.

Stein et al, recently discovered another transcript with potential clinical relevance, Metastasis-Associated in Colon Cancer-1 MACC [37]. MACC1 has a regulatory role in the HGF/Met signaling pathway which has an important role in cell migration, invasion, and metastatic potential [38]. MACC1 expression in the primary tumor and in plasma of CC patients was shown to be an independent risk factor for metastasis [38,39]. The prognostic significance of CCAT1 is remains unclear. We are in the process of studying a large cohort of patients with early CC for level of CCAT1 expression, and will correlate expression of this transcript with overall survival.

Serum markers in clinical use for CC (CEA and CA 19-9) are neither sensitive nor specific [40]. Therefore the most common application of CEA and CA-19-9 is to monitor patients for recurrent disease following treatment of CC or to monitor response to systemic therapy [41]. If the measurement of CCAT1 levels in the plasma of CC patients should prove both feasible and reproductive, then it may be added to the current serum markers to monitor disease behavior and patient response to treatment.

Another interesting observation is that CCAT1 expression is higher in patients with peritoneal metastasis originating from colon cancer compared to peritoneal surface malignancy of appendiceal origin. The results did not reach statistical significance in this particular comparison, due to the large variability of transcript expression observed in the colon cancer patients. Nevertheless, we think that further investigation is warranted because appendiceal adenocarcinoma, as do some colon adenocarcinomas, demonstrates preferential spread to the peritoneal surface rather than to solid visceral organs.

The expression of CCAT1 in tissues of all stages of the adenoma-carcinoma sequence of colorectal cancer together with our previous preliminary observations [22] that CCAT1 can be amplified from the blood and stool samples of patients with CRC point to a promising, novel biomarker for CRC. CCAT1 can be used to enhance pathological staging in borderline cases by *in-situ* hybridization; it can be used in an RNA-based stool assay for the screening and early detection of CRC, and in blood tests for the diagnosis and follow-up of CRC patients.

In summary, we studied CCAT1 expression in human biospecimens spanning the biological spectrum of benign, pre-malignant and malignant colonic tissues and demonstrated CCAT1 up-regulation, which peaked in tissues from adenomas and colon adenocarcinoma lymph node metastases.

## Conclusions

We conclude that CCAT1 is up-regulated in the colon adenoma-carcinoma sequence. This up-regulation is evident in pre-malignant conditions and through all disease stages, including advanced metastatic disease suggesting a role in tumorigenesis and the metastatic process.

## Abbreviations

CC: Colon cancer; CCAT1: Colon cancer associated transcript-1; RNA: Ribonucleic acid; qRT-PCR: Quantitative reverse-transcriptase polymerase chain reaction; ISH: *In situ* hybridization; RQ: Relative quantity; RDA: Representational difference analysis; RACE: Rapid amplification of cDNA ends; IRB: Institutional review board; UICC: Union for international cancer control; AJCC: American joint committee on cancer; GAPDH: Glyceraldehyde 3-phosphate dehydrogenase; TBST: Tris-buffered saline tween-20; FITC: Fluorescein isothiocyanate; BCIP: 5-Bromo-4-chloro-3-indolyl phosphate; LN: Lymph node; USMCI: United states military cancer institute

## Competing interests

The authors declare that they have no competing interests.

## Authors’ contributions

Conception and design: AN, AOG. Acquisition of data: BA, NE, HP, MI, MR, VP,VT, DH, AOG, AN. Analysis and interpretation of data: AS, DH, BT, AOG, AN. Drafting of manuscript: BA, AS, AOG, AN. Critical revision: BA, NE, HP, MI, MR, VP, VT, AS, BT, DH, AOG, AN. Supervision: AS, AN, DH, AOG. We certify that all individuals who qualify as authors have been listed; each has participated in one or more of the following areas: conception and design of this work, the acquisition and/or analysis of data, the writing, and/or critical revision of the document, and supervision of this cooperative research effort. All contributing authors approve of the submission of this version of the manuscript and assert that the document represents valid work. If information derived from another source was used in this manuscript, we obtained all necessary approvals to use it and made appropriate acknowledgements in the document. All contributing authors take public responsibility for this work. All contributing authors have no disclosures to make. All authors read and approved the final manuscript.

## Pre-publication history

The pre-publication history for this paper can be accessed here:

http://www.biomedcentral.com/1471-2407/13/196/prepub

## Copyright protection

Some of the contributing authors are military service members (or employees of the U.S. Government: AS), and this work was prepared as part of their official duties. Title 17 U.S.C. 105 provides the “Copyright protection under this title is not available for any work of the United States Government.” Title 17 U.S.C. 101 defines a U.S. Government work as a work prepared by a military service member or employee of the U.S. Government as part of that person’s official duties.
